# Dyschromatosis universalis hereditaria with *SASH1* mutation improved with picosecond laser treatment

**DOI:** 10.1093/skinhd/vzaf024

**Published:** 2025-04-22

**Authors:** Liyan Yuan, Ying Luo, Chao Yang, Mao-Qiang Man, Bin Yang, Zhenfeng Liu

**Affiliations:** Dermatology Hospital, Southern Medical University, Guangzhou, People’s Republic of China; Dermatology Hospital, Southern Medical University, Guangzhou, People’s Republic of China; Dermatology Hospital, Southern Medical University, Guangzhou, People’s Republic of China; Dermatology Hospital, Southern Medical University, Guangzhou, People’s Republic of China; Dermatology Hospital, Southern Medical University, Guangzhou, People’s Republic of China; Dermatology Hospital, Southern Medical University, Guangzhou, People’s Republic of China

## Abstract

Dyschromatosis universalis hereditaria (DUH) is a rare autosomal dominant pigmentary skin disorder characterized by hypo- and hyperpigmented macules over the body. Although DUH is associated with mutations in *ABCB6* and *SASH1*, other factors also contribute to the pathogenesis of DUH, as the lesions typically appear on the exposed areas of the skin and do not develop in all individuals with *SASH1* mutations. Most reported cases of *SASH1* mutations are in Chinese or Japanese patients who do not require treatment. Herein, we report a rare case of an 11-year-old boy presenting with an 8-year history of widespread brown spots. The lesions, which began on his face and spread to the trunk, limbs and oral mucosa, developed without photosensitivity. Whole-exome sequencing helped identify a heterozygous *SASH1* mutation (c.1529G > A; exon13, NM_015278.5). Initial treatment with intense pulsed light did not result in any improvement; however, subsequent picosecond laser treatment led to significant improvement. Hence, this case highlights the phenotypic variability of DUH associated with *SASH1* mutations and the potential role played by additional genetic or environmental factors in disease expression. Furthermore, picosecond laser treatment may be effective against hyperpigmented lesions, although further studies are required to assess its long-term efficacy and safety.

## Teaser

We identified a novel pathogenic mutation in SASH1 in a Chinese boy with DUH, thus further expanding the mutation spectrum of this gene in DUH. We demonstrated that picosecond laser treatment is a safe and effective method of treating hyperpigmented lesions in patients with DUH.

Dyschromatosis universalis hereditaria (DUH) is a rare pigmentary genodermatosis, which is typically inherited in an autosomal dominant manner. It is characterized by multiple pinpoint- to pea-sized hypo- and hyperpigmented macules in a reticular pattern. Skin lesions often develop during the first few years of life. Mutations in at least two genes, *ABCB6* and *SASH1*, contribute to the aetiology of DUH.^1^

Here, we report a rare case of DUH with a heterozygous mutation in *SASH1* and provide a literature review.

## Case report

**Figure 1:**
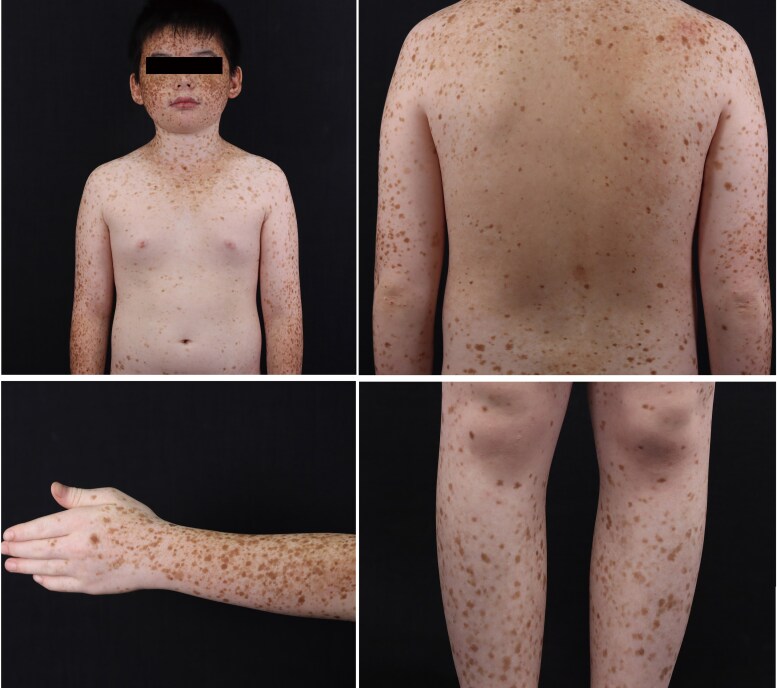
Clinical pictures of the patients.

**Figure 2:**
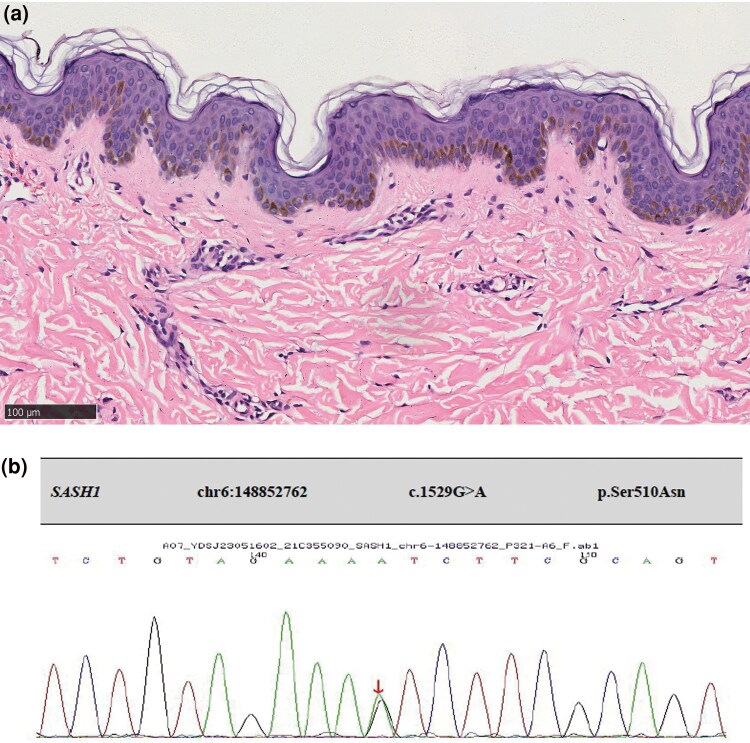
Histology and whole-exome sequencing. (a) Haematoxylin and eosin staining of skin lesion (scale bar = 100 mm). (b) Whole-exome sequencing result.

An 11-year-old boy presented with an 8-year history of widespread brown spots on his body. The patient was born to nonconsanguineous parents and delivered vaginally at full term. Asymptomatic brown spots developed on the face at approximately 3 years of age and gradually spread to the trunk and limbs, without hypopigmentation spots. The spots predominantly appeared in sun-exposed areas and involved mucosal surfaces. The patient reported no photosensitivity, with the results of UVA-minimal erythema dose (MED): 25.4 J cm^−2^, and UVB-MED: 44.6 mJ cm^−2^. Physical examination revealed densely distributed brown spots on the face, trunk, limbs and labial mucosa (Figure 1). Histological examination of a biopsy from the hyperpigmented macules revealed a normal epidermis, excessive blue-grey keratinization, significant increase in pigment in the basal layer, and sparse perivascular lymphocytic infiltration in the superficial dermis (Figure 2a). Whole-exome sequencing performed on samples obtained from the patient and his family members (father, mother and younger brother) revealed a heterozygous mutation in *SASH1*, c.1529G > A (exon13, NM_015278.5) (Figure 2B), resulting in a p.Ser510Asn missense mutation. The family members (father, mother and brother) of the patient did not carry this mutation. The diagnosis of hereditary DUH^1^ was established.

**Figure 3:**
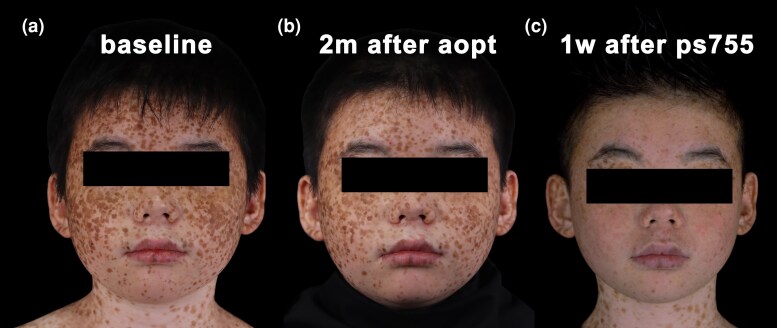
Clinical pictures before and after the treatment. (a) Before the treatment; (b) 2 months after the treatment with Intense Pulsed Light; (c) 1 week after the treatment with picosecond laser.

Initial treatment with intense pulsed light (560 nm cutoff filter, 17 J/cm^2^, two pulses, and pulse delay of 35 ms) (Lumenis M22, Yokneam, Israel) led to erythema and crust formation at the treatment sites immediately after application. However, no significant improvement was observed after 2 months. The patient subsequently underwent treatment with a picosecond laser (Cynosure Inc., Westford, MA, USA) at a spot size of 3 mm, fluence of 2.83 J/cm^2^ and pulse width of 750 ps. Significant improvement was observed 1 week post-treatment (Figure 3). Despite the positive outcome, the patient declined further treatment owing to concerns about medical costs.

## Discussion

Since the first reported case of DUH in 1933,^2^ more than 100 cases have been reported worldwide. Of these, more than three-quarters have been reported from Japan, India, China and other Asian countries.^3^ The prevalence of DUH is estimated to be 0.3 per 100 000.^2^ The incidence is slightly higher in women than in men.^4^ However, 15 out of 22 *SASH1* mutation-related cases occur in men (Table 1).^1,3,5–14^ The mechanisms underlying these sex-related differences remain unclear.

**Table 1:** *SASH1* mutation-associated dyschromatosis

Phenotype	Country/Region	Sex	Age (yr)	Age of onset	Family history	Involved sites	Mutation site	Treatment and outcome	Ref.
DUH	China	M	11	3	No	Whole body and mucosa	c.1529G > A	Picosecond laser is effective	Present case
DUH	China	M	22	4	yes	The whole body except the palms, soles and mucosa.	c.1529G > A	—	Zhong WL, *et al*. 2019^13^
DUH	China	No detail information was given in the paper			yes	No detailed information was given in the paper	c.2000G > A	—	Wu N, *et al*. 2020^10^
							c.2019T > C		
							c.2126T > G		
DUH	China	F	25	3	yes	The trunk, face, neck and limbs	c.1784T > C	—	Shellman YG, *et al*. 2015^8^
DUH	China	M	42	7 months	yes	The whole body except the palmoplantar and mucosal areas	c.1651T > C	—	Shellman YG, *et al*. 2015^8^
DUH	China	F	6	2	yes	The whole body except the palms, soles and oral mucosa	c.1553A > C		Zhang J, *et al*. 2016^12^
DUH	China	M	26	1	yes	The abdomen, back, limbs, face, and neck	c.1547G > A	—	Nogita T, *et al*. 2011^15^
DUH	China	F	Young*	infant	yes	The whole body	c.1547G > T	—	Nogita T, *et al*. 2011^15^
DUH	China	M	39	12	yes	The whole body.	c.1761C > G,	—	Cui H, *et al*. 2020^3^
DUH	China	M	30	1	yes	The limbs, trunks, face, and neck	c.1757T > C	Laser (not specified which laser) and the outcome)	Murthy AB, *et al*. 2023^16^
lentiginous phenotype	China	M	7	3	Yes	The trunk and face, elbow joints and dorsal area of hands and feet.	c.1537A > C	—	Araki Y, *et al*. 2021^5^
lentiginous phenotype	China	M	15	—	No	The face, trunk, extremities and mucosa	1527_1530dupAAGT	—	Araki Y, *et al*. 2021^5^
lentiginous phenotype	China	M	27	18 months	yes	The whole body	c.1519T > G	a 755-nm Q-switched alexandrite laser is effective	Courcet JB, *et al*. 2015^6^
lentiginous phenotype	Japan	M	3	14 months	No	The face, limbs and trunk.	c.1758C > G,	—	Liu JW, *et al*. 2021^7^
lentiginous phenotype	Japan	F	27	2	yes	The face, limbs and trunk.	c.1592C > A		Liu JW, *et al*. 2021^7^
lentiginous phenotype	Japan	F	16	3	yes	The face, limbs and trunk.	c.1930C > T	—	Liu JW, *et al*. 2021^7^
lentiginous phenotype	Japan	F	4	2	No	The face, limbs and trunk.	c.1574C > G	—	Liu JW, *et al*. 2021^7^
lentiginous phenotype	Japan	M	3	8 months	No	The face, limbs and trunk.	c.1547G > T	—	Liu JW, *et al*. 2021^7^
lentiginous phenotype	Japan	M	38	4	yes	The face, limbs and trunk.	c.1930C > T	—	Liu JW, *et al*. 2021^7^
lentiginous phenotype	Morocco	M	32	1	yes	The face, trunk, extremities with palmoplantar keratoderma, alopecia and brittle teeth.	c.1849G > A	—	Yang Y, *et al*. 2023^11^
lentiginous phenotype	Morocco	F	39	6 months	yes	On the dorsal hands and feet, and face, with spinocellular carcinomas, dry soles and palms with desquamation, alopecia and dystrophic nails.	c.1849G > A	—	Yang Y, *et al*. 2023^11^
lentiginous phenotype	USA	M	Adult	first decade of life	yes	The whole body, but prominent in sun-exposed areas.	c.1556 G > A	—	Wang J, *et al*. 2017^9^

DUH is a generalized pigmentary dermatosis characterized by numerous, asymptomatic, well-demarcated, hyper-or hypopigmented macules in a reticular pattern on the trunk and extremities. Skin lesions develop before the age of 6 years in 18% of patients; approximately 20% of the patients have dyspigmentation at birth.^15^ It typically manifests at an early age and affects almost the entire body, including nails, hair and teeth; however, they are usually not present in the palms and soles. Facial lesions occur in approximately 50% of patients.^4^ Some patients may also show extracutaneous manifestations, including deafness, visual impairment and neurological symptoms.^16^ The disease severity does not change with the seasons or spontaneously regress with age.^4^

DUH is typically inherited in an autosomal dominant (AD) pattern with variable penetrance, although autosomal recessive patterns and sporadic cases have been reported.^15^ Based on different linkage regions, including 6q24.2-q25.2, 12q21-q23 and 2q35, DUH can be classified into three types: DUH1 [Online Mendelian Inheritance in Man (OMIM 127500), DUH2 (OMIM 612715), and DUH3 (OMIM 615402)]. DUH 1 and DUH 3 exhibit AD inheritance, whereas DUH 2 follows an autosomal recessive pattern.^1^

DUH is primarily characterized by an increase in melanosome synthesis or activity, rather than a defect in melanocyte numbers. *ABCB6* and *SASH1* have been identified as pathogenic genes associated with DUH. SASH1, a member of the SLy family of signal adapter proteins, acts as a candidate tumour suppressor and plays a regulatory role in the tumorigenesis of breast and colon cancers.^17,18^ In pigmentation disorders such as DUH, SASH1 promotes melanocyte transepithelial migration through a Gαs–SASH1–IQGAP1–E-cadherin-dependent pathway^14^ or via a p53-POMC-MC1R signalling cascade, thereby enhancing the phosphorylation of ERK1/2 and CREB and resulting in a hyperpigmented phenotype.^19^

Heterozygous SASH1 missense mutations have been associated with pigmentation disorders, including multiple lentiginous phenotypes and DUH. Analysis of genotype–phenotype correlation in patients with DUH or lentiginous phenotypes relies mainly on previously published reports. Several heterozygous missense mutations in *SASH1* have been identified in patients with dyschromatosis. *SASH1*-associated skin dyschromia includes classic DUH and the lentiginous phenotype; it predominantly involves sun-­exposed areas with or without dyschromatosis or concomitant palmoplantar keratoderma and skin carcinoma.^20^ One patient with DUH and a *SASH1* mutation (c.1529G > A) presented with lentigines on the face and hands, which gradually spread to the trunk and extremities, without the involvement of the palms, soles, and mucosa. Sun exposure exacerbated the skin lesions,^1^ suggesting that sunlight acts as a triggering factor.

Notably, individuals with identical mutation sites may not exhibit the same phenotype. The current patient with a *SASH1* mutation (c.1529G > A) showed oral mucosal involvement, whereas other patients with the same mutation lacked oral mucosal lesions.^1^ Moreover, individuals with *SASH1* mutations may not develop DUH. In a previous report, a father and son shared the same mutation site (c.1930C > T); however, only the father had DUH.^5^ These findings suggest that there are additional factors, apart from genetic ones, which contribute to DUH pathogenesis.

An effective treatment for DUH has not yet been established. Traditional therapeutic approaches for pigmentary lesions include surgical excision, dermabrasion, electrodesiccation and chemical peeling.^15^ However, adverse effects such as scarring or dyspigmentation limit the use of these methods in generalized pigmentary disorders such as DUH. Although hyperpigmented macules have been successfully treated with Q-switched alexandrite laser,^15^ the long-term efficacy remains uncertain. Narrow-band ultraviolet B therapy is effective in some patients;^21^ however, given the association between *SASH1* mutations and skin cancer, the risk of skin carcinoma must be considered.^18^ In the present study, we demonstrated that picosecond laser treatment is a safe and effective method of treating hyperpigmented lesions in a patient with DUH. However, further studies are necessary to evaluate the long-term efficacy and safety of picosecond laser treatment in a large cohort of patients with DUH.

In summary, we successfully identified a novel pathogenic mutation in *SASH1* in a Chinese boy with DUH, thus further expanding the mutation spectrum of this gene in DUH. Multiple mutation sites in *SASH1* are associated with DUH; however, these sites do not necessarily determine phenotype. Sunlight and other factors may trigger or exacerbate DUH. Lasers, such as 755-nm Q-switched alexandrite and picosecond lasers, offer effective treatment options. However, larger cohort trials are required to validate the efficacy and safety of picosecond lasers for DUH.

## Data Availability

The data underlying this article are available upon request from the corresponding author.
